# Symptom-based stratification of patients with primary Sjögren's syndrome: multi-dimensional characterisation of international observational cohorts and reanalyses of randomised clinical trials

**DOI:** 10.1016/S2665-9913(19)30042-6

**Published:** 2019-09-25

**Authors:** Jessica R Tarn, Nadia Howard-Tripp, Dennis W Lendrem, Xavier Mariette, Alain Saraux, Valerie Devauchelle-Pensec, Raphaele Seror, Andrew J Skelton, Katherine James, Peter McMeekin, Shereen Al-Ali, Katie L Hackett, B Clare Lendrem, Ben Hargreaves, John Casement, Sheryl Mitchell, Simon J Bowman, Elizabeth Price, Colin T Pease, Paul Emery, Peter Lanyon, John Hunter, Monica Gupta, Michele Bombardieri, Nurhan Sutcliffe, Costantino Pitzalis, John McLaren, Annie Cooper, Marian Regan, Ian Giles, David Isenberg, Vadivelu Saravanan, David Coady, Bhaskar Dasgupta, Neil McHugh, Steven Young-Min, Robert Moots, Nagui Gendi, Mohammed Akil, Bridget Griffiths, Svein J A Johnsen, Katrine B Norheim, Roald Omdal, Deborah Stocken, Colin Everett, Catherine Fernandez, Leeds CTRU, John D Isaacs, Jacques-Eric Gottenberg, French ASSESS cohort, Valerie Devauchelle-Pensec, Philippe Dieude, Jean Jacques Dubost, Anne-Laure Fauchais, Vincent Goeb, Eric Hachulla, Claire Larroche, Véronique Le Guern, Jacques Morel, Aleth Perdriger, Xavier Puéchal, Stephanie Rist, Damien Sen, Jean Sibilia, Olivier Vittecoq, Joelle Benessiano, Sarah Tubiana, Karine Inamo, Stanie Gaete, Djilali Batouche, Domitille Molinari, Mickael Randrianandrasana, Isabelle Pane, Adeline Abbe, Gabriel Baron, Philippe Ravaud, Jacques-Eric Gottenberg, Philippe Ravaud, Xavier Puéchal, Véronique Le Guern, Jean Sibilia, Vincent Goeb, Claire Larroche, Jean-Jacques Dubost, Stéphanie Rist, Alain Saraux, Valérie Devauchelle-Pensec, Jacques Morel, Gilles Hayem, Pierre Hatron, Aleth Perdriger, Damien Sene, Charles Zarnitsky, Djilali Batouche, Valérie Furlan, Joelle Benessiano, Elodie Perrodeau, Raphaele Seror, Xavier Mariette, S Brown, N Coy Navarro, C Pitzalis, P Emery, S Pavitt, J Gray, C Hulme, F Hall, R Busch, P Smith, L Dawson, M Bombardieri, W-F Ng, C Pease, E Price, N Sutcliffe, C Woods, S Ruddock, C Everett, C Reynolds, E Skinner, A Poveda-Gallego, J Rout, I Macleod, S Rauz, S Bowman, Wan-Fai Ng, UK Primary Sjögren's Syndrome Registry, Wan-Fai Ng, Simon J Bowman, Bridget Griffiths, Frances Hall, Elalaine C Bacaba, Helen Frankland, Robert Moots, Kuntal Chadravarty, Shamin Lamabadusuriya, Michele Bombardieri, Constantino Pitzalis, Nurhan Sutcliffe, Celia Breston, Nagui Gendi, Karen Culfear, Claire Riddell, John Hamburger, Andrea Richards, Saaeh Rauz, Sue Brailsford, Joanne Dasgin, Joanne Logan, Diarmuid Mulherin, Jacqueline Andrews, Pau Emery, Alison McManus, Colin Pease, David Pickles, Alison Booth, Marian Regan, Jon King Kin, Amanda Holt, Theodoros Dimitroulas, Lucy Kadiki, Daljit Kaur, George Kitas, Abdul Khan, Tracey Cosier, Panthakalam, Kell Mintrim, Mark Lloyd, Lisa Moore, Esther Gordon, Cathy Lawson, Monica Gupta, John Hunter, Lesley Stirton, Gill Ortiz, Elizabeth Price, Suzannah Pelger, Claire Gorman, Balinder Hans, Gavin Clunie, Suzanne Lane, Ginny Rose, Sue Cuckow, Michael Batley, Ruby Einosas, Susan Knight, Deborah Symmons, Beverley Jones, Andrew Carr, Suzanne Edgar, Francisco Figuereido, Heather Foggo, Dennis Lendrem, Iain Macleod, Sheryl Mitchell, Christine Downie, Jessica Tarn, James Locke, Shereen Al-Ali, Sarah Legg, Kamran Mirza, Ben Hargreaves, Laura Hetherington, Adrian Jones, Peter Lanyon, Alice Muir, Paula White, Steven Young-Min, Susan Pugmire, Saravanan Vadivelu, Annie Cooper, Marianne Watkins, Anne Field, Stephen Kaye, Devesh Mewar, Patricia Medcalf, Pamela Tomlinson, Debbie Whiteside, Neil McHugh, John Pauling, Julie James, Andrea Dowden, Mohammed Akil, Jayne McDermott, Olivia Godia, David Coady, Elizabeth Kidd, Lynne Palmer, Charles Li, Sarah Bartrum, De Mead, Bhaskar Dasgupta, Victoria Katsande, Pamela Long, Olivia Godia, Erin Vermaak, Janet Turner, Usha Chandra, Kirsten MacKay, Stefano Fedele, Ada Ferenkeh-Koroma, Ian Giles, David Isenberg, Helena MaConnell, Nyarko Ahwiren, Stephen Porter, Paul Allcoa, John McLaren

**Affiliations:** a Faculty of Medical Sciences, Newcastle University, Newcastle upon Tyne, UK; b Newcastle-upon-Tyne Hospitals NHS Foundation Trust, Newcastle upon Tyne, UK; c Université Paris-Sud, AP-HP Université Paris-Saclay, Hôpital Bicêtre, Department of Rheumatology, INSERM UMR1184, Le Kremlin-Bicêtre, France; d Lymphocytes B et auto-immunité, Inserm U1227, University of Brest, Brest, France; e Centre Hospitalier Régional Universitaire de Brest, Brest, France; f Interdisciplinary Computing & Complex BioSystems Research Group, School of Computing, Newcastle University, Newcastle upon Tyne, UK; g Faculty of Health and Life Science, Northumbria University, Newcastle upon Tyne, UK; h Department of Pathological Analyses, College of Science, University of Basrah, Basrah, Iraq; i National Institute for Health Research Newcastle In Vitro Diagnostics Co-operative, NewcastleUniversity, Newcastle upon Tyne, UK; j University Hospital Birmingham, Birmingham, UK; k Great Western Hospitals NHS Foundation Trust, Swindon, UK; l Biomedical Research Centre, Leeds Teaching Hospitals NHS Trust Leeds, Leeds, UK; m Nottingham University Hospitals NHS Trust, Rheumatology, Derby Road, Nottingham, UK; n Gartnavel General Hospital, Glasgow, UK; o Centre for Experimental Medicine & Rheumatology, William Harvey Research Institute, Barts and The London School of Medicine & Dentistry, Queen Mary University of London, London, UK; p Barts Health NHS Trust, London, UK; q NHS Fife, Kirkcaldy, UK; r Royal Hampshire County Hospital, Winchester, UK; s University Hospitals of Derby and Burton, Derby, UK; t Centre for Rheumatology, University College London, London, UK; u Gateshead Health NHS Foundation Trust, Gateshead, UK; v City Hospitals Sunderland NHS Foundation Trust, Sunderland, UK; w Southend University Hospital NHS Foundation Trust, Westcliff-on-Sea, UK; x Department of Pharmacy and Pharmacology, University of Bath, Bath, UK; y Portsmouth Hospitals NHS Trust, Portsmouth, UK; z University Hospital Aintree, University of Liverpool, Liverpool, UK; aa Basildon and Thurrock University Hospitals NHS Foundation Trust, Basildon, UK; ab Sheffield Teaching Hospitals NHS Foundation Trust, Sheffield, UK; ac Helse Stavanger HF, Stavanger, Norway; ad Clinical Trials Research Unit, Leeds Institute of Clinical Trials Research, University of Leeds, Leeds, UK; ae Department of Rheumatology, Centre de Référence National Pour les Maladies Auto-Immunes Systémiques Rares, CNRS, Strasbourg, France; af Institut de Biologie Moléculaire et Cellulaire, Immunopathologie et Chimie Thérapeutique, Université de Strasbourg, Strasbourg, France

## Abstract

**Background:**

Heterogeneity is a major obstacle to developing effective treatments for patients with primary Sjögren's syndrome. We aimed to develop a robust method for stratification, exploiting heterogeneity in patient-reported symptoms, and to relate these differences to pathobiology and therapeutic response.

**Methods:**

We did hierarchical cluster analysis using five common symptoms associated with primary Sjögren's syndrome (pain, fatigue, dryness, anxiety, and depression), followed by multinomial logistic regression to identify subgroups in the UK Primary Sjögren's Syndrome Registry (UKPSSR). We assessed clinical and biological differences between these subgroups, including transcriptional differences in peripheral blood. Patients from two independent validation cohorts in Norway and France were used to confirm patient stratification. Data from two phase 3 clinical trials were similarly stratified to assess the differences between subgroups in treatment response to hydroxychloroquine and rituximab.

**Findings:**

In the UKPSSR cohort (n=608), we identified four subgroups: Low symptom burden (LSB), high symptom burden (HSB), dryness dominant with fatigue (DDF), and pain dominant with fatigue (PDF). Significant differences in peripheral blood lymphocyte counts, anti-SSA and anti-SSB antibody positivity, as well as serum IgG, κ-free light chain, β2-microglobulin, and CXCL13 concentrations were observed between these subgroups, along with differentially expressed transcriptomic modules in peripheral blood. Similar findings were observed in the independent validation cohorts (n=396). Reanalysis of trial data stratifying patients into these subgroups suggested a treatment effect with hydroxychloroquine in the HSB subgroup and with rituximab in the DDF subgroup compared with placebo.

**Interpretation:**

Stratification on the basis of patient-reported symptoms of patients with primary Sjögren's syndrome revealed distinct pathobiological endotypes with distinct responses to immunomodulatory treatments. Our data have important implications for clinical management, trial design, and therapeutic development. Similar stratification approaches might be useful for patients with other chronic immune-mediated diseases.

**Funding:**

UK Medical Research Council, British Sjogren's Syndrome Association, French Ministry of Health, Arthritis Research UK, Foundation for Research in Rheumatology.

**Video Abstract:**

Symptom-based stratification of patients with Sjögren's syndrome

## Introduction

Primary Sjögren's syndrome is a chronic, immune-mediated inflammatory disease, characterised by ocular and oral dryness, musculoskeletal pain, profound fatigue, and an increased risk of lymphoma.^1^ Symptom severity varies greatly between individuals; some report unbearable pain and dryness, some report debilitating fatigue, but others report few symptoms.^2^ Co-existing clinical features, such as anxiety and depression, are common and might modulate symptoms of primary Sjögren's syndrome.^3^, ^4^, ^5^ No effective treatment exists, and the direct and indirect health costs of primary Sjögren's syndrome are substantial.^6^, ^7^

A key challenge in the development of therapy for patients with primary Sjögren's syndrome and many other immune-mediated inflammatory diseases is heterogeneity in clinical presentation, presumably driven by differences in underlying molecular pathology and responsible, at least partially, for variable responses to therapies.^8^ Precision medicine refers to approaches to optimally define disease complexity and heterogeneity in order to tailor therapies to the appropriate patient populations and subpopulations. Although stratified medicine has had considerable success in oncology, progress has been slower in immune-mediated inflammatory diseases. Existing approaches attempt to identify disease subgroups on the basis of biological heterogeneity. Unlike cancers, in which the clinical endpoints and target tissue are well defined, the clinical manifestations of many immune-mediated inflammatory diseases, including primary Sjögren's syndrome, are diverse. For some clinical manifestations, such as fatigue and pain, the target tissue is unclear. Consequently, the relationship between dysregulated biological pathways and clinical symptoms is often difficult to establish. This heterogeneity also poses challenges in defining the appropriate clinical endpoints with which to measure effectiveness of therapies in clinical trials.^9^, ^10^, ^11^

**Research in context**

**Evidence before this study**

We searched MEDLINE for “Sjögren's Syndrome”, including the terms “subsets”, “sub-groups”, “phenotypes”, and “endotypes”, filtering by “clinical trial”, “stratification”, and “immune-mediated inflammatory”. We also included major review articles from noted experts. We identified numerous reports of clinical heterogeneity, but weak evidence of biological subsets, mainly from small studies without external validation data and without clinically meaningful stratification.

**Added value of this study**

This study shows the existence of subgroups of patients with primary Sjögren's syndrome with distinct profiles of symptom severity, clinical and biological profiles and therapeutic responses. Our symptom-based stratification approach not only represents a novel approach to stratified medicine but also an original strategy linking pathobiology to symptomatology, which is often poorly understood in clinical medicine.

**Implications of all the available evidence**

These data have important implications for future research, clinical practice, trial design, and therapeutic development. First, our data open new research avenues to further explore the pathogenesis of this disease. Second, our findings that different patient subgroups appear to respond differently to hydroxychloroquine and rituximab might help with personalised care of these patients. Furthermore, our symptom-based stratification enables stratification and clinical management plans to be made quickly, without the requirement for sophisticated laboratory analyses. Finally, our proposed stratification tool might have a substantial effect on clinical trial design, in terms of patient stratification and corresponding clinical study endpoints, improving efficiency and reducing the cost of drug development.

In this study, we aimed to develop a symptom-based stratification approach and test its clinical, biological, and therapeutic significance.

## Methods

### Study design and participants

The UK Primary Sjögren's Syndrome Registry (UKPSSR) is a national observational cohort of clinically well-characterised patients with primary Sjögren's syndrome, who fulfil the 2002 American European Consensus Group (AECG) classification criteria. Our study cohort was recruited between Aug 13, 2009 and Sept 27, 2011.^12^ The UKPSSR is an ongoing initiative. The UKPSSR holds detailed clinical and laboratory data that is collected prospectively, including patient-reported symptoms collected using standardised questionnaires.

Two independent European cohorts of patients with primary Sjögren's syndrome were used as validation cohorts: the French Assessment of Systemic Signs and Evolution of Sjögren's Syndrome (ASSESS) cohort, and the Norwegian Stavanger cohort. All patients met the 2002 AECG criteria. The ASSESS cohort is a French cohort of patients recruited from 15 tertiary centres for autoimmune diseases between 2006 and 2009. The Stavanger cohort consists patients with primary Sjögren's syndrome attending the Stavanger University Hospital (Norway), recruited between January, 2014, and August, 2016.

Baseline data that permitted our symptom-based stratification were available from two randomised, placebo-controlled trials of patients with primary Sjögren's syndrome; the JOQUER trial,^17^ which investigated hydroxychloroquine treatment, and the TRACTISS trial,^18^ which investigated rituximab treatment. In the JOQUER trial, 120 patients were randomly assigned (56 to hydroxychloroquine and 64 to placebo), of whom 107 had baseline data permitting symptom-based stratification. In the TRACTISS trial, 133 patients were randomly assigned (67 to rituximab and 66 to placebo), of whom 114 had baseline data permitting stratification.

Research ethics approval was granted by the UK North-West Research Ethics Committee, and ethics committees in France (Bichat Teaching Hospital ethics committee) and Norway (Regional Ethics Committee West [2010/1455]). All participants provided informed consent.

### Cluster discovery and model development

Symptom-based subgroups were first identified by hierarchical cluster analysis of the severity of five common symptoms of primary Sjögren's syndrome (pain, fatigue, dryness, anxiety, and depression) in the UKPSSR cohort. Pain, fatigue, and dryness were measured using the EULAR Sjogren's Syndrome Patient Reported Index (ESSPRI),^13^ anxiety and depression using the Hospital Anxiety and Depression Scale (HADS).^14^ The ESSPRI and HADS have been extensively validated with excellent internal consistency and test-retest reliability.^13^, ^14^

Clustering analysis is sensitive to sample size^15^ and both external validation cohorts were small compared with the UKPSSR. Therefore, we used a simple algorithm (the Newcastle Sjögren's Stratification Tool [NSST]) permitting classification of individual patients into our four candidate subgroups in the clinic. A multinomial logistic regression model was developed, predicting cluster membership on the basis of the same five patient reported symptoms using a training subset of two-thirds of all patients (selected randomly); the remaining third was used for testing of the model. The receiver operating characteristic curves for the training and test datasets had an area under the curve of greater than 0·95 for all symptom-based subgroups (appendix p 8), indicating that the NSST stratification algorithm faithfully replicates the initial clustering model. The NSST was used to assign patients from the independent cohorts to symptom-based subgroups for validation of the key clinical and biological findings. The NSST is available to academic and non-commercial researchers as an Excel macro algorithm.

### Endotype discovery

All clinical and laboratory (including transcriptomic) parameters, as well as peripheral blood samples, for analyses in this study for all three cohorts were obtained at the time of recruitment and entry to the respective cohorts.

Ocular dryness was assessed using Schirmer's test and oral dryness using unstimulated salivary flow (USF). Systemic disease activity was measured using the EULAR Sjögren's Syndrome Disease Activity Index (ESSDAI).^13^ Other clinical and demographic parameters assessed are listed in the appendix (p 3-4).

Data on serum β2-microglobulin, and κ-free and λ-free light-chains (FLCs) were available for 596 UKPSSR patients, and CXCL13 data were available for 112 patients. Data on these serum proteins were available from the ASSESS cohort. Serum protein data were log-transformed before ANOVA testing for significant differences across symptom-based subgroups (appendix p 6).

Whole-blood transcriptomic data for 196 UKPSSR and 312 ASSESS patients were available (appendix p 6). Data were mean centred, and module activity scores calculated according to the Chaussabel method.^16^ These transcriptomic modular data were used for discriminant analysis with the wide linear method maximising discrimination between symptom-based subgroups and estimating canonical scores for each patient by singular value decomposition of the standardised data. We also investigated differences in expression of individual annotated Chaussabel modules between the symptom-based subgroups using ANOVA across subgroups and cohorts.

### Statistical analysis

A schematic representation of the cohorts and analysis stages is shown in the appendix (p 7). All statistical tests and graphical rendering were done using the R statistical package and SAS JMP statistical data visualisation software (version 14). Summary data—including medians and quartiles, or percentages for counts data—are presented, and Kruskal-Wallis analysis of ranks, or ANOVA of rank or log-transformed data, were used to identify potential differences between symptom-based subgroups. For the comparison of cohorts, linear models were constructed including terms for subgroups and cohort and their interaction. For the JOQUER and TRACTISS trials, we reanalysed trial outcomes including terms for subgroup and treatment and their interaction.

### Role of the funding source

The funders and sponsors of the study had no role in study design, data collection, data analysis, data interpretation, or writing of the report. WFN, DWL, and JRT had full access to all the data in the study. WFN and JDI had the final responsibility for the decision to submit for publication.

## Results

Clinical and demographic characteristics of the UKPSSR cohort (n=608), and the ASSESS (n=334) and Stavanger (n=62) validation cohorts, are summarised in the appendix (p 14). Patients were predominantly female and white. There were no significant differences in age, median ESSDAI, or ESSPRI scores between the three cohorts. The disease duration was longer in the Stavanger cohort than in the UKPSSR and ASSESS cohorts. We found small but statistically significant differences in body-mass index (BMI) between the three cohorts (appendix p 14).

**Figure 1:**
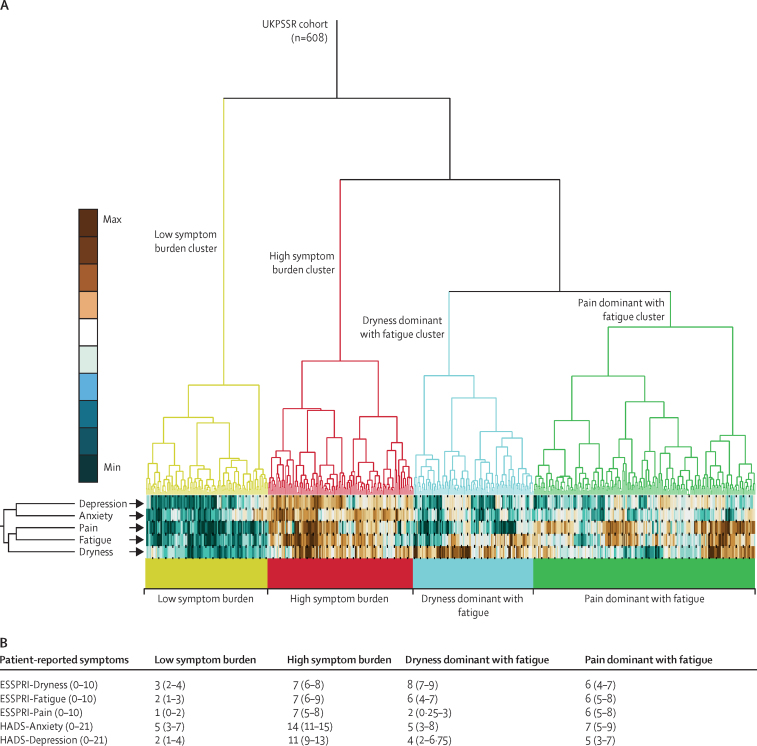
Patient reported symptom scores for each cluster (A) Dendogram and symptom heatmap shows results of the cluster analysis of patient symptom scores from the ESSPRI and HADS scores for pain, fatigue, dryness, anxiety, and depression. The symptoms are colour-coded in the heatmap at the base of the dendrogram: teal is low, white is intermediate, and brown represents a high symptom score. (B) Median (IQR) patient reported symptom scores within each cluster. ESSPRI=the EULAR Sjogren's Syndrome Patient Reported Index. HADS=Hospital Anxiety and Depression Scale.

An unsupervised hierarchical cluster analysis using the patient-reported symptoms of pain, fatigue, dryness, anxiety, and depression identified four key clusters (figure 1). These clusters were low symptom burden (LSB), high symptom burden (HSB), dryness dominant with fatigue (DDF), and pain dominant with fatigue (PDF).

The LSB cluster was characterised by low scores on all five symptoms whereas the HSB cluster scored highly on all five symptoms. The key features of the DDF cluster were high dryness and fatigue scores, but low anxiety and depression scores. The PDF cluster was characterised by high pain and fatigue scores but low anxiety and depression scores. We then used multinomial logistic regression to develop a stratification tool on the basis of observed cluster membership, for stratification of other patients with primary Sjögren's syndrome into these four symptom-based subgroups.

We found no statistically significant differences in age, sex, disease duration, and ESSDAI, nor in prescription of hydroxychloroquine, prednisolone, or other immunosuppressive drugs, between different subgroups.

In the UKPSSR cohort we found significant differences in salivary flow; Schirmer's test, serum IgG, lymphocyte counts, and the prevalence of anti-SSA and anti-SSB antibody positivity between these four subgroups (table 1). The DDF subgroup showed the lowest values on objective measures of glandular function. The LSB subgroup had the highest serum IgG levels and low lymphocyte counts. Anti-SSA and anti-SSB antibody positivity was higher in the DDF and LSB subgroups than in the HSB and PDF subgroups (table 1).

**Table 1:** Objective parameters between subgroups

	**Low symptom burden**	**High symptom burden**	**Dryness dominant with fatigue**	**Pain dominant with fatigue**	**p value**
**Unstimulated salivary flow, ml/15 min**					
UKPSSR	0·4 (0·0–1·05)	0·2 (0·0–1·0)	0·1 (0·0–0·8)	0·3 (0·0–1·2)	0·0097
ASSESS	0·3 (0·1–1·7)	0·4 (0·1–2·2)	0·0 (0·0–0·2)	0·2 (0·1–1·5)	<0·0001
Stavanger	1·7 (0·4–2·2)	0·8 (0·1–2·5)	0·2 (0·0–0·7)	0·9 (0·0–1·7)	0·12
Combined	0·3 (0·1–1·5)	0·3 (0·0–1·3)	0·1 (0·0–0·2)	0·3 (0·0–1·4)	<0·0001
**Schirmer's Test (mm/5 mins)**					
UKPSSR	3·0 (0·0–6·8)	3·0 (0·5–10·5)	2·0 (0·0–6·5)	4·0 (0·1–10·5)	0·014
ASSESS	5·3 (2·0–15·0)	5·8 (2·3–14·4)	7·0 (0·0–13)	7·5 (3·5–15)	0·26
Stavanger	7·0 (2·5–13·6)	6·8 (2·8–23·1)	1·5 (0·0–4·5)	5·5 (2·5–14·5)	0·024
Combined	3·9 (0·5–9·0)	5·0 (1·0–12·5)	2·3 (0.0–7·1)	5·0 (2.0–12·5)	<0·0001
**Lymphocytes, ×10**^9^**/L**					
UKPSSR	1·2 (1.0–1·6)	1·5 (1·2–1·8)	1·3 (1.0–1·7)	1·3 (1·0–1·7)	<0·0001
ASSESS	1·3 (1.0–1·8)	1·5 (1·1–1·7)	1·2 (1.0–1·6)	1·5 (1·1–1·8)	0·025
Stavanger	1·4 (0·7–1·7)	1·9 (1·6–2·4)	1·2 (0·9–1·4)	1·8 (1·3–2.0)	0·030
Combined	1·3 (1.0–1·6)	1·5 (1·2–1·7)	1·2 (1.0–1·7)	1·4 (1·1–1·8)	<0·0001
**Serum IgG, mg/dL**					
UKPSSR	18·0 (14·5–22·9)	14·1 (11·1–18·2)	16·6 (13.0–20·9)	14·4 (11·1–19·5)	<0·0001
ASSESS	15·0 (12·3–18·7)	12·8 (10·7–16·7)	15·2 (11·1–20·6)	12·5 (9·8–16·1)	0·0028
Stavanger	14·0 (10·7–16.0)	11·1 (9·3–11·9)	14·9 (12·4–18)	11·7 (10·0–13·1)	0·0054
Combined	16·6 (13·2–21·5)	13·4 (10·7–17.0)	16·0 (12·4–20·3)	13·1 (10·2–17·7)	<0·0001
**SSA or SSB positive, or both, n/N (%)**					
UKPSSR	111/119 (93·3%)	128/147 (87·0%)	113/120 (94·2%)	189/222 (85·1%)	0·024
ASSESS	45/64 (70·0%)	50/85 (58·8%)	32/43 (74·4%)	76/141 (53·9%)	0·049
Stavanger	9/10 (90·0%)	3/5 (60·0%)	18/18 (100%)	17/29 (58·6%)	0·0018
Combined	165/193 (85·5%)	181/237 (76·4%)	163/181 (90·0%)	282/392 (71·9%)	<0·0001
**Patients, n (%)**					
UKPSSR (N=608)	119 (19·6%)	147 (24·2%)	120 (19·7%)	222 (36·5%)	..
ASSESS (N=334)	64 (19·2%)	85 (25·4%)	43 (12·9%)	142 (42·5%)	..
Stavanger (N=62)	10 (16·1%)	5 (8·1%)	18 (29·0%)	29 (46·8%)	..
Combined (N=1004)	193 (19·2%)	237 (23·6%)	181 (18·0%)	393 (39·1%)	..

Data are median (IQR), unless otherwise indicated. The combined probability is for the pooled analysis across all three cohorts after rank transformation and ANOVA including subgroup, cohort, and subgroup by cohort interaction. For p values corrected for multiple comparisons see appendix (p 15). UKPSSR=UK Primary Sjögren's Syndrome Registry. ASSESS=Assessment of Systemic Signs and Evolution of Sjögren's Syndrome.

These clinical differences observed in the UKPSSR were assessed in two independent cohorts using the NSST. We found statistically significant differences in the expected direction for IgG, lymphocyte counts, anti-SSA anti-SSB positivity, and consistent differences in objective measures of oral and ocular glandular function (table 1).

CXCL13, β2-microglobulin, and κ-FLC concentrations differed significantly across subgroups in the UKPSSR cohort, with the highest concentrations occurring in the DDF subgroup (table 2). The DDF subgroup had the highest prevalence of lymphoma within the UKPSSR cohort (table 2). Subgroup differences in serum levels of these proteins were comparable in the ASSESS cohort and followed a similar pattern to those in the UKPSSR cohort (table 2).

**Table 2:** Serum protein analysis

	**Low symptom burden**	**High symptom burden**	**Dryness dominant with fatigue**	**Pain dominant with fatigue**	**p value**
**κ-free light chains (mg/L)**					
UKPSSR	3·20 (0·52)	3·06 (0·52)	3·25 (0·6)	3·08 (0·47)	0·0336
ASSESS	2·80 (0·55)	2·72 (0·59)	2·89 (0·64)	2·59 (0·56)	0·0106
λ-free light chains (mg/L)					
UKPSSR	2·94 (0·45)	2·86 (0·47)	3·00 (0·50)	2·86 (0·44)	0·0485
ASSESS	2·75 (0·52)	2·69 (0·51)	2·78 (0·65)	2·63 (0·52)	0·3375
β2-microglobulin (mg/L)					
UKPSSR	1·34 (0·22)	1·30 (0·24)	1·38 (0·27)	1·32 (0·24)	0·0336
ASSESS	1·20 (0·22)	1·16 (0·19)	1·25 (0·24)	1·12 (0·20)	0·0031
CXCL13 (pg/ml)					
UKPSSR*	4·86 (0·53)	4·97 (0·63)	5·33 (0·68)	5·00 (0·70)	0·040
ASSESS	4·74 (1·00)	4·93 (1·12)	4·98 (0·78)	4·48 (0·83)	0·0010
Lymphoma prevalence, n/N (%)					
UKPSSR	2/119 (1·7%)	12/208 (5·8%)	13/120 (10·8%)	6/222 (2·7%)	0·0113

Data are mean (standard error), unless otherwise indicated. Data were available for 596 patients from the UKPSSR and 334 patients from the ASSESS cohorts. p values are for analysis of variance of log-transformed protein data testing separately for differences between subgroups within the two cohorts. Percentages are shown for lymphoma prevalence from the UKPSSR. The p value for lymphoma prevalence is for an exact permutation test for differences in prevalence between the subgroups. UKPSSR=UK Primary Sjögren's Syndrome Registry. ASSESS=Assessment of Systemic Signs and Evolution of Sjögren's Syndrome.

* CXCL13 was measured for a subset of 112 patients in the UKPSSR only (appendix p 16).

Discriminant analysis was done using the available transcriptomics data from the UKPSSR and ASSESS cohorts. The appendix (p 10) shows the three-dimensional positions of individual patients’ overall transcriptomic modular profiles in canonical space based upon the first three canonical variables (video). The overall transcriptomic profiles of the four subgroups were consistent between these two independent cohorts.

**Figure 2:**
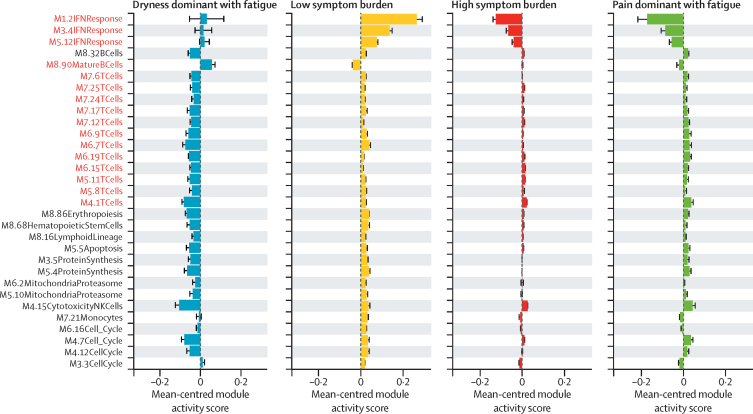
Chaussabel module activity scores Chaussabel module activity scores adjusting for batch differences for the UKPSSR and ASSESS transcriptomics datasets centred on the mean for each module. Shown are the top 31 modules with significant differences between subgroups in the UKPSSR and ASSESS cohorts. Negative values imply inhibition and positive values imply activation. The error bars represent 95% CIs. For example, the first module is activated in patients in the dryness dominant with fatigue and low symptom burden subgroups, but inhibited in patients in the high symptom burden. and pain dominant with fatigue subgroups. Of particular interest are the modules IFN Response, mature B cells, and T cells (highlighted red). Unadjusted p values were used in this analysis. Details of the modules including the adjusted p values are listed in the appendix (p 17). IFN=interferon.

At the level of individual transcriptomic modules, we found statistically significant differences in 31 (31%) of 100 annotated scores for Chaussabel transcriptomic module activity between the subgroups (figure 2). Scores for interferon (IFN) module activity were significantly higher in the LSB and DDF subgroups than in the HSB and PDF subgroups. The LSB subgroup had the highest activity score for most modules except the mature B-cell modules. By contrast, the DDF subgroup had the lowest score for most modules apart from the IFN and mature B-cell modules, which was highest in the DDF subgroup. The differences in the mature B-cell modules are largely driven by the altered expression of genes associated with B cell signalling, germinal centres, lymphoproliferative disease, and oxidative stress.

When we attempted to use ESSDAI to stratify patients according to their systemic disease activity, the stratification based on patient reported symptoms outperformed the ESSDAI-based stratification, giving better goodness-of-fit and identifying more Chaussabel modular differences (appendix pp 4–5).

To assess the therapeutic significance of our stratification strategy, we used the NSST algorithm to stratify patients from the JOQUER and TRACTISS trials. In the TRACTISS trial, because rituximab is known to target B cells, we hypothesised that the DDF subgroup was more likely to respond to rituximab than were the other subgroups because the DDF subgroup had the highest mature B-cell transcriptomic modular score, serum κ-FLC and λ-FLC, β2-microglobulin, and CXCL13 concentrations.

**Figure 3:**
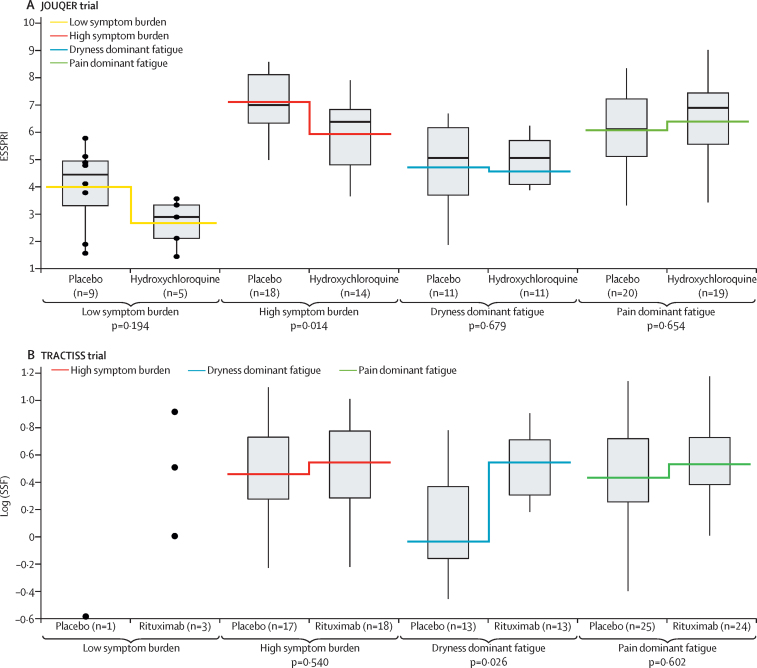
Reanalysis of two clinical trials using symptom-based subgroups (A) ESSPRI scores for each subgroup for patients in placebo and HCQ groups in the JOQUER trial.^17^ Box plots show the median ESSPRI scores, quartiles, and ranges for placebo and hydroxychloroquine for LSB, HSB, DDF, and PDF subgroups. The step break indicates the mean ESSPRI scores of the placebo and hydroxychloroquine treatments for each subgroup. Although we found no overall treatment effect, we found a significant treatment by subgroup interaction. This consistency test is statistically significant (p=0·036). The p values shown are for the contrast within each subgroup. (B) Stimulated salivary flow for each subgroup for patients in the placebo and rituximab groups of the TRACTISS trial.^18^ Box plots of log transformed data show the median SSF and ranges for placebo and rituximab treatments for each subgroup. Data are shown for the LSB subgroup; however, statistical analysis was not done because of insufficient data in this stratum. Although the figures show group values at the end of the trial, the probability values refer to the statistical analysis on changes from baseline as per the original clinical protocols. LSB=low symptom burden. HSB=high symptom burden. DDF=dryness dominant with fatigue. PDF=pain dominant with fatigue. SSF=stimulated salivary flow.

Consistent with the original studies, we found no statistically significant overall (ie, non-stratified) treatment effect for the change in ESSPRI scores in either study group. However, we found treatment–subgroup interactions in both studies (figure 3). Individual contrasts were formed to estimate treatment differences within each subgroup (figure 3). In the JOQUER trial, we found a clinically significant reduction (ie, improvement) in ESSPRI scores (ie, >1)^19^ for the HSB subgroup (p=0·01). In the TRACTISS trial, we found no statistically significant effects on ESSPRI scores, but as hypothesised, patients in the DDF subgroup receiving rituximab had significantly higher USF (p=0·04) and stimulated salivary flow (figure 3, p=0·03) than did patients in the DDF subgroup who were on placebo at week 48. No treatment effect was observed for other subgroups in either trial.

## Discussion

Using patient reported symptoms, we identified four subgroups of patients with primary Sjögren's syndrome with distinct clinical and biological profiles, suggesting that these are true endotypes,^19^ and these patients are likely to differ in their response to targeted therapies. Our data support the use of these subgroups in dissecting the biological basis of this complex disease and its associated debilitating symptoms, informing clinical management and the design of future clinical trials of patients with primary Sjögren's syndrome. To our knowledge, this is the first report showing distinct subsets of an immune-mediated inflammatory disease and linking clinical and pathobiological heterogeneity, with direct clinical implications.

Conventionally, patients with primary Sjögren's syndrome are thought to consist of two subtypes—those with predominantly glandular symptoms and those with extra-glandular (systemic) manifestations. However, many patients do not fit neatly into these subtypes and no consensus criteria exist for such classification.

Our stratification approach centred on identifying, characterising, and validating clinical subgroups before attempting to assess underlying pathobiological heterogeneity. Our rationale was that a clinical phenotype in an immune-mediated inflammatory disease is likely to be underpinned by networks of dysregulated biological pathways rather than one or a few pathways. Consequently, without initial careful clinical characterisation, even the most advanced methods for analysing high-dimensional data face a daunting task.^20^, ^21^

For our initial exploratory clustering analysis, alongside the cardinal primary Sjögren's syndrome symptoms (pain, fatigue, dryness),^19^ anxiety and depression were included because these symptoms are common in patients with primary Sjögren's syndrome, affect overall symptom burden (particularly pain and fatigue), and are relevant for clinical trial outcomes.^3^, ^5^ Furthermore, immune-mediated mechanisms have been implicated in depression,^22^ raising the possibility that depression might be a manifestation of the pathobiology of primary Sjögren's syndrome. Patient-reported data play an increasingly recognised role in clinical trials, therapeutic licensing, and health-care policy decisions, and are key to capturing quality of life and health economic outcomes.^23^ Although some researchers might have concerns over the subjectivity and reliability of patient reported symptoms, the instruments we used to measure symptom severity have been shown to have excellent internal consistency and test-retest reliability.^13^, ^14^ Furthermore, the symptom profiles between the four subgroups that we describe differ markedly from one another and therefore the risk of misclassification due to variability in self-reported assessments is small. Importantly, had our data not been robust, validation in two independent cohorts would have been highly unlikely. From a clinical perspective, our approach allows patient stratification at the point of care. More importantly, symptoms are the key driver for patients with primary Sjögren's syndrome seeking medical help and hence the use of health-care resources.

The LSB and DDF subgroups shared many objectively measured laboratory features, including reduced lymphocyte counts and increased IgG concentrations, and were more likely to be anti-SSA and anti-SSB positive than were the HSB and PDF subgroups. However, as anticipated, the DDF subgroup had the poorest objectively measured glandular function. Furthermore, we found differences in the transcriptomic modular profiles between the LSB and DDF subgroups. Lymphoma prevalence was also highest in the DDF subgroup in the UKPSSR cohort. Consistently, the DDF subgroup had the highest serum concentrations of CXCL13 (which has been linked to lymphoma in patients with primary Sjögren's syndrome^24^), the highest levels of β2-microglobulin (a prognostic marker of poor outcomes in malignant lymphoma^25^), and altered expression of genes associated with B-cell signalling, germinal centres, lymphoproliferative disease, and oxidative stress. Long-term studies, however, are needed to substantiate the association of the DDF subgroup with lymphoma development. That the LSB subgroup had the highest levels of IFN and T cell transcriptomic modular activities might seem counterintuitive. However, our findings are consistent with the results of a study by Bodewes and colleagues, showing that pain scores are reduced in patients with primary Sjögren's syndrome that show systemic IFN activity.^26^ Similarly, in a model of IFN-α induced fatigue, Russell and colleagues showed that persistent fatigue is not associated with peripheral immune activation.^27^ Collectively, these data highlight our poor understanding of the mechanisms underpinning the symptomatology of primary Sjögren's syndrome and challenge the simple notion of peripheral immune activation being responsible for the symptoms.

We found no significant differences between the PDF and HSB subgroups in objectively measured laboratory parameters. The overall transcriptomic profiles differed considerably between these groups, however, despite similarities in the level of transcription of individual modules. Furthermore, clinical responses to hydroxychloroquine appeared to differ between these groups. Further characterisation of the pathobiology of these two subgroups is warranted, including non-immunological mechanisms.

Whole-blood transcriptomics might not correlate with protein expression in either blood or target tissue.^28^, ^29^ However, in related conditions, such as systemic lupus erythematosus, transcriptomic changes in blood are similar to those in target organs such as the kidney.^29^ Also, mass cytometry data have shown that cellular components in blood correlate with clinical parameters and glandular inflammation in patients with primary Sjögren's syndrome.^30^ Because the target organ for many of the debilitating symptoms (such as pain and fatigue) of primary Sjögren's syndrome is unknown, blood is a reasonable starting point in the search for biological differences between symptom-based subgroups.

Although alternative transcriptomics analytical approaches**,** including Kyoto Encyclopedia of Genes and Genomes, BioCarta, and Ingenuity Pathway Analysis exist, gene sets from these databases derive from multiple data sources and tissue types and might not be relevant for whole-blood analyses. Furthermore, the same gene might appear in multiple pathways and gene sets, making inferring which pathways are disturbed difficult. Instead, we used Chaussabel modules to explore the pathobiological differences between groups. These modules were developed in a data-driven manner, specifically for the characterisation of transcriptomic profiles in human whole-blood samples, such that each gene set (module) is mutually exclusive.

From a therapeutic perspective, the biological differences between the four subgroups might inform future stratified approaches. For instance, therapies targeting T cells might be effective for the LSB subgroup but not DDF subgroup, whereas treatments targeting CXCL13 or B cells might be the preferred strategy for the DDF subgroup. On the other hand, therapies targeting interferon pathways could be more effective for DDF and LSB subgroups, although careful selection of clinical endpoints to measure therapeutic responses for these subgroups is crucial, particularly for the LSB subgroup, in which symptoms were minimal.

Our data also support close consideration of these symptom-based subgroups when designing future clinical trials of primary Sjögren's syndrome. For example, results from the JOQUER and TRACTISS trials were initially disappointing. However, reanalysis of the data stratifying by these subgroups indicated treatment effects in response to hydroxychloroquine for the HSB subgroup and to rituximab for the DDF subgroup. We expected to find treatment effects in response to rituximab for the DDF subgroup, given that the DDF subgroup had the highest mature B-cell modular scores. The original trials were not powered for a stratified analysis, however, and further validation of these findings is needed. Our data also suggest that the choice of primary endpoint in future trials might differ between symptom-based subgroups and will be crucial in assessing the outcome of interventions. Additionally, although data on anxiety and depression have not been routinely collected in clinical trials of primary Sjögren's syndrome (and indeed in most patient cohorts), we would like to see our stratification algorithm incorporated into future trial designs. We have not presented longitudinal data but the study of long-term outcomes and stability of these subgroups of patients with primary Sjögren's syndrome is ongoing. Our preliminary analysis suggests that the subgroups are largely stable for 4–5 years.

This study is not without limitations. Although clustering analysis is a useful exploratory tool, the method is sensitive to the choice of clustering method and assessment metrics, and there is no consensus on the optimal approach. In this study, we adopted a team approach, involving biostatisticians, bioinformaticians, data scientists, and clinicians in decision-making. In selecting the clustering method to use for the subsequent development of a stratification tool, we considered clinical experience as well as statistical assessment of cluster metrics (appendix pp 1–3). We believe that our choice to use hierarchical clustering analysis was vindicated by the successful validation against two external, independent cohorts. A small subgroup possibly exists within the patients in the PDF subgroup with very high pain, fatigue, and dryness score (appendix p 2), and a future study to investigate this possibility might be worthwhile. A further limitation of our work is that, although we have painstakingly combined data from multiple platforms, cohorts, and trials, none of these studies was designed with stratification as the primary objective. In particular, the trials were not powered for a stratified analysis. Furthermore, although the transcriptomics and cytokine data are illuminating, these data were not collected to address biomarker selection or stratification objectives.

We do not exclude other stratification strategies. However, our data show that symptom-based stratification is a robust and clinically meaningful approach, addressing the clinical heterogeneity of patient experience and reflecting differences in pathobiological profiles and therapeutic responses.

## Data sharing

We are happy to share the linked anonymised data derived from the UKPSSR upon request. Transcriptomic data for the UKPSSR cohort have been deposited in the ArrayExpress database at EMBL-EBI, under access number E-MTAB-8277. All requests should be made to W-FN, and data sharing will be subject to the terms of the UKPSSR data sharing agreement to ensure all users of the data adhere to the legal requirements of using personal data.

## Supplementary Materials

Supplementary appendix

Supplementary video Projection of patient data in canonical space following discriminant analysis of the four subgroups using all annotated Chaussabel module activity scores.
