# Supplementary material for: Symptom-based stratification of patients with primary Sjögren's syndrome: multi-dimensional characterisation of international observational cohorts and reanalyses of randomised clinical trials
Source: Lancet Rheumatol. 2019 Sep 25;1(2):e85–94. doi: 10.1016/S2665-9913(19)30042-6 (PMC7134527; doi:10.1016/S2665-9913(19)30042-6)
Supplement: Supplementary appendix [file mmc1.pdf]

THE LANCET
Rheumatology

Supplementary appendix

This appendix formed part of the original submission and has been peer reviewed. We post it as supplied by the authors.

Supplement to: Tarn JR, Howard-Tripp N, Lendrem DW, et al. Symptom-based stratification of patients with primary Sjögren's syndrome: multi-dimensional characterisation of international observational cohorts and reanalyses of randomised clinical trials. *Lancet Rheumatol* 2019; **1:** e85–94.

For the video mentioned on page 10, please visit the following website: https://youtu.be/kR7fb--o5wo.

Appendix

A) Additional Statistical Information on Methods

The statistical methods include: initial exploratory data analyses using clustering of the UKPSSR discovery cohort; development of the Newcastle Sjogren's syndrome Stratification tool (NSST) using multinomial regression based on the initial clustering approach (which is the stratification model that we subsequently validate using independent cohorts); linear modelling of subgroup differences across discovery and validation cohorts; and re-analyses of two phase III clinical trials, testing for differences between treatments and placebo controls for the different subgroups. We used clustering analysis on patient-reported symptoms to identify patients with similar symptomology, tested for heterogeneity in biological and clinical variables between those clusters, developed a classification tool permitting patients from independent cohorts to be assigned to symptom-based subgroups, tested whether those same biological and clinical effects were replicated in those independent cohorts, before going on to use the classification tool to stratify patients from two historical clinical trials testing for differences in therapeutic response (see Supplementary Figure S1).

1. We used two validated instruments to collect Patient Reported Outcome data [1-4]:

a) Pain, fatigue and dryness were measured using EULAR Sjogren's Syndrome Patient Reported Index (ESSPRI) which consists of 3 domains: Pain, Fatigue and Dryness. Each domain uses a numeric scale of 0-10 indicating "No symptom" to "Worst imaginable symptom". The reliability of ESSPRI is supported by the study by Seror R et al [2]. In this study, 395 patients (from 15 countries) were asked to complete the ESSPRI questionnaire two days apart. The intra-rater correlation coefficient was excellent (0.94 (CI: 0.89, 0.97)). A difference of one unit on the ESSPRI is considered of clinical significance and it is accepted to be the response criteria for clinical studies in pSS.

b) Anxiety and depressive symptoms were measured using Hospital Anxiety and Depression scale (HADS). HADS was developed in 1983 as a screening instrument for use in hospital outpatient departments (the same setting of the three cohorts) and has been extensively used in many studies. In a systematic review performed in 2000, of 747 studies [2], the mean Cronbach's alpha (a measure of internal consistency) for HADS-Anxiety and HADS-Depression scores were very good (0.83 and 0.82 (CI: 0.68-0.93 and 0.67-0.90 respectively)).

Therefore, these two instruments are appropriate tools for the evaluation of these five symptom domains.

2. All statistical tests and graphical rendering were performed using the R statistical package, SAS and SAS JMP Statistical Data Visualization software[5-7]. Except where stated we used a probability value of $p<0.05$ as the threshold for all statistical significance tests. Otherwise we used a FDR-adjusted Benjamini-Hoechberg probability value threshold of $p<0.05$.

3. In the exploratory data analysis during the discovery phase of this study, the initial clusters – based on symptom scores for patients in the UKPSSR – were identified using Ward's hierarchical clustering method. The decision to use hierarchical clustering was supported by our Analytical Team (comprising statisticians (DWL, BCL), bioinformaticians (AJS, JC), data scientists (JRT) as well as clinicians (WFN, JDI and the majority of the co-authors)). Hierarchical Clustering assumes a hierarchy of symptoms that is consistent with the clinical expectations of the relationships between the clusters. Furthermore, Ward's method performs well in the absence of multivariate outliers and with relatively low dimensional hierarchical data (in this case only 5 dimensions). Using the cubic clustering criterion, Ward's method supported up to five candidate clusters, while alternative metrics such as the pseudo-t^2 supported just two clusters.

Ultimately, four clusters were selected by the Analytical Team based upon heat maps of the severity scores of the five common pSS symptoms (ESSPRI-pain, ESSPRI-fatigue, ESSPRI-dryness, HADS-anxiety and HADS-depression). As shown in the dendrogram and heatmap of Figure 1A (and below), a 2-cluster model would yield the LSB cluster, with the remaining patients (i.e. those from the HSB, DDF and PDF groups) being classified as a single cluster which consists of a subgroup with highly heterogeneous levels of symptoms. Given that the LSB subgroup is least likely to seek medical intervention, the 2-cluster stratification model has limited clinical utility. A 3-cluster model would identify the HSB cluster, as well as the LSB cluster, with the third cluster consisting of the DDF and PDF patients, a heterogeneous group of patients with low levels of symptoms of anxiety and depression but variable levels of symptoms of pain, fatigue or dryness. In contrast, the 4-cluster model identified an additional subgroup with severe symptoms of dryness but minimal symptoms of pain. We excluded a fifth candidate cluster – a subset of PDF patients with low levels of anxiety and depression and high levels of pain, fatigue and dryness - because of its small size. For similar reasons the Analytical Team decided against models with higher numbers of clusters. Therefore, the 4-cluster model was taken forward for validation against independent cohorts.

Figure 1A (modified) illustrated the group membership for two to five cluster models.

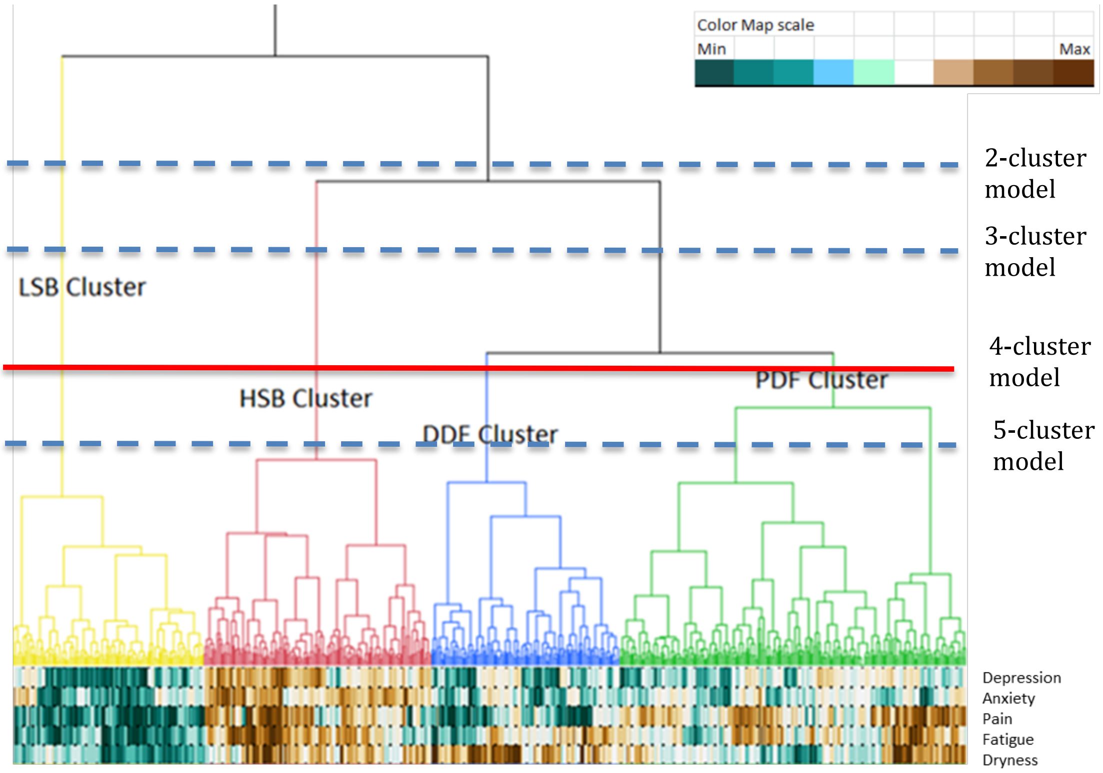

[**LSB**=Low Symptom Burden; **HSB**=High Symptom Burden; **DDF**=Dryness Dominant Fatigue; **PDF**=Pain Dominant Fatigue]

4. Given the uncertainties inherent in clustering analysis, and in particular the choice of number of clusters, we later repeated the clustering exercise using a variety of alternative clustering methods. In particular, interest centred on the choice of the number of candidate clusters. These methods included centroids, nearest neighbours, k-means, Gaussian mixtures and more modern methods including Markov consensus clustering. The optimal number of clusters was explored using Cubic Clustering Criteria, Pseudo-F and Pseudo-t^2 statistics, AIC, BIC and other metrics as appropriate for the method. These additional clustering approaches generally favoured fewer clusters: while all of the

clustering methods identified a low symptom burden cluster, the breakdown of the remaining patients was more nuanced. The decision to take forward four stratification subgroups was taken in the knowledge that these could be collapsed in the event that they were not clinically useful. The fact that we were able to recapitulate the clinical and biological findings in independent cohorts based on the four-cluster model provides a level of confidence in this decision. That symptom-based subgroups were effective in identifying biologically and clinically important differences which were subsequently validated in two external, independent cohorts, indicates that our stratification approach is robust.

5. Rather than using the clustering formula directly, we chose to create a simple classification tool allowing us – and other researchers – to assign patients to subgroups based upon patient reported symptom scores. Having already used unsupervised clustering methods to identify clusters, we were then able to switch to supervised methods predicting cluster membership. We considered using the original clustering formula for classification, but took the view that clinicians in our field are probably more familiar with logistic and multinomial regression as classification tools. Since we had now identified candidate clusters, we were able to build a supervised multinomial regression model for this purpose[8]. This model predicted membership of the four clusters based on ESSPRI and HADS scores for a randomly selected, two-thirds training set of the UKPSSR cohort. The model predicted cluster membership based upon Pain, Fatigue, Dryness, Anxiety and Depression scores. We then tested this model using the remaining, one-third hold-out data set. We compared predicted cluster membership using the classification tool, to the actual observed cluster membership for each patient in the hold-out set. We estimated ROC AUC values for the four subgroups (see *Supplementary Figure S2* below). The model performed well in the hold-out validation set. Model estimates and standard errors are available in the Excel stratification workbook (see *Supplementary Figures S2B and S4* below). This model was then used as a tool to classify patients into the four symptom-based subgroups for the independent validation observational cohorts and JOQUER and TRACTISS clinical trial cohorts. Note that this approach permitted stratification of patients from independent cohorts and historical clinical trials. Recapitulation of the biological and clinical findings between subgroups in independent cohorts and trials lends strong support to the notion that these represent biologically and clinically meaningful subgroups underpinned by pathobiological differences.

The NSST is available to academic/non-commercial researchers as an Excel macro algorithm to stratify pSS patients based on these five key symptoms into the **LSB**, **DDF**, **HSB** and **PDF** subgroups. The NSST can be accessed via the following weblink: https://github.com/SJOGRENS/Symptom-Based-Subgroups. Note that the users are required to obtain a password from the Analytic team and to confirm that the use of the algorithm is for non-commercial research projects.

6. Summary data – including either medians and quartiles, or means and standard deviations for continuous data, or percentages for counts data – are presented for clinical and laboratory variables. Kruskal-Wallis analysis of ranks – an omnibus test for overall group heterogeneity – permitted screening of clinical and laboratory variables for differences between subgroups. For discrete data, contingency table methods were used and chi-square or exact permutation tests computed testing for differences between subgroups. In particular, tests for differences in the prevalence of lymphoma are based on exact, permutation-based methods rather than large sample approximations. Please note that the Kruskal-Wallis analysis is an omnibus test of overall heterogeneity between subgroups. Note that, rather than attempting to control false discoveries through p-value adjustments, we instead took forward the full list of prospective candidates parameters (see below) to the next stage for validation in the independent cohorts.

Basic clinical and demographic parameters that were recorded include age, sex, body mass index (BMI), AECG duration, ESSDAI and ESSPRI. The 18 candidate clinical variables were: Haemoglobin, Total white cell count, Neutrophils, Lymphocytes, Platelet, ESR, CRP, Thyroid stimulation hormone, Complement component 3, Complement component 4, IgG, IgA, IgM, Ro/La

positivity, Schirmer's, unstimulated salivary flow, lymphoma prevalence*, EQ-5D*. The 8 candidate serum protein variables were κ-FLC, λ-FLC, combined FLC, BAFF, β2m, CXCL13, IL-17*, TNF-α*. [*denotes variables which were not available from the independent cohorts for validation]. We have also included the corresponding p-values after Benjamini-Hochberg FDR corrections in Supplementary Tables S2.1 and S2.2.

For the analysis of transcriptomics for which the multiplicity problem is more substantial, the candidate list needed to be reduced. Here we either performed Benjamini-Hochberg adjustments when testing individual modules or constructed canonical variables for discriminant analysis. Note that in the main text, we used unadjusted p-values when comparing individual annotated transcriptomic modules between subgroups.

7. For comparison of cohorts, linear models were constructed using rank-transformed variables including terms for Subgroup, Cohort and their interaction: Subgroup x Cohort. The Subgroup x Cohort interaction test was used as a 'consistency' test checking that the differences between Subgroups were consistent across the Cohorts. In addition, linear models were constructed to analyse log-transformed serum proteins, permitting the estimation of differences between subgroups, cohorts, and their interaction. Again, the interaction term was used to assess 'consistency' between subgroups across the cohorts.

8. We performed discriminant analysis on the Chaussabel module scores, plotting the location of each patient in canonical space (see supplementary Figure S3). Canonical variables are linear combinations of the individual module scores permitting discrimination of the phenotypes after adjustment for differences between cohorts. In addition, these Canonical variables are multivariate normally distributed and orthogonal to each other. Each phenotype has a multivariate centroid and measurable variability around that centroid. This permits statistical testing for differences between phenotypes in transcriptomic space and calculation of multivariate confidence intervals[9]. Canonical plots are presented – together with the 50% ellipsoids about the centroid of each sub-group – separately for the UKPSSR and ASSESS cohorts. The 50% density ellipsoids are calculated as the multivariate mean, or centroid, ± 0.6 multivariate standard deviations. 50% is the default in many systems as it captures the location of the bulk of the data without obscuring their relative separation. While the entropy R2 for the discriminant model is 69% and the ROC AUC for each of the subgroups is greater than 0.90, we avoided making exaggerated claims for this model and instead use the results to simply visualize the separation and relative locations of the four phenotypic subgroups in transcriptome space for the two cohorts.

9. In order to compare the performance of symptom-based stratification and stratification by disease activity, we stratified the UKPSSR and ASSESS data using the EULAR Sjögrens Syndrome Disease Activity Index (ESSDAI). This separates patients with Low, Moderate and High systemic disease activity (ESSDAI scores 0-4, 5-13, ≥14 respectively). We found that stratification by symptoms (i.e. using the NSST) out-performed stratification by disease activity. The NSST stratification identifies more clinical and biologically meaningful differences. In addition we compared the goodness of fit of the two stratification models by comparing the residual deviances after fitting the NSST-based and ESSDAI-based strata to the transcriptomics data. The residual deviance was consistently minimized for the NSST-based stratification model compared to the ESSDAI-based model. The NSST-based model gave a better model fit than the ESSDAI-based model on 70 of the 100 Chaussabel modules (binomial test, p = 0.00037). In addition, we repeated the analysis using a four-level ESSDAI categorization – inactive (0), low (1-4), moderate (5-13), high (14 or above). This means that the number of groups is equal and the degrees of freedom identical for model comparison. This slightly reduces the number of modules

for which the NSST-based stratification shows the better fit – from 70 to 68 of the 100 modules (binomial test, p=0.0004).

10. For the JOQUER and TRACTISS trials, we analysed the primary outcome variable– change in ESSPRI scores from baseline to the end of the trial – including terms for Subgroup and Treatment and their interaction: Subgroup x Treatment. We then formed contrasts to compare Treatments within each Subgroup. For the TRACTISS trial there was no statistically significant effect on the change in ESSPRI scores from baseline to the end of the trial. However, there were significant differences for the secondary outcomes – unstimulated and stimulated salivary flow, USF and SSF respectively. USF and SSF were bounded by zero and positively skewed. Accordingly, analyses were performed on log transformed data: $\log_{10}(USF+k)$ and $\log_{10}(SSF+k)$ where k is a small constant ($k=10^{-2}$). There were statistically significant differences in both USF and SSF in the **DDF** subgroup. In the JOQUER trial there were clinically important differences in ESSPRI for both the **HSB** and **LSB** subgroups – though only the former was statistically significant.

References for the Appendix:

1. Seror R, Ravaud P, Mariette X, Bootsma H, Theander E, Hansen A, Ramos-Casals M, Dörner T, Bombardieri S, Hachulla E, Brun JG, Kruize AA, Praprotnik S, Tomsic M, Gottenberg JE, Devauchelle V, Devita S, Vollenweider C, Mandl T, Tzioufas A, Carsons S, Saraux A, Sutcliffe N, Vitali C, Bowman SJ; EULAR Sjögren's Task Force. EULAR Sjogren's Syndrome Patient Reported Index (**ESSPRI**): development of a consensus patient index for primary Sjogren's syndrome. Ann Rheum Dis. 2011 Jun;70(6):968-72.
2. Seror R, Theander E, Brun JG, Ramos-Casals M, Valim V, Dörner T, Bootsma H, Tzioufas A, Solans-Laqué R, Mandl T, Gottenberg JE, Hachulla E, Sivils KL, Ng WF, Fauchais AL, Bombardieri S, Valesini G, Bartoloni E, Saraux A, Tomsic M, Sumida T, Nishiyama S, Caporali R, Kruize AA, Vollenweider C, Ravaud P, Vitali C, Mariette X, Bowman SJ; EULAR Sjögren's Task Force. Validation of EULAR primary Sjögren's syndrome disease activity (ESSDAI) and patient indexes (**ESSPRI**). Ann Rheum Dis. 2015 May;74(5):859-66.
3. Herrmann C. International experiences with the Hospital Anxiety and Depression Scale: a review of validation data and clinical results. Journal of Psychosomatic Research 1997; 42(1):17-41.
4. Bjelland I, Dahl AA, Haug TT, Neckelmann D. The validity of the Hospital Anxiety and Depression Scale. An updated literature review. Journal of Psychosomatic Research 2002; 52(2):69-77.
5. R Core Team (2017). R: A language and environment for statistical computing. R Foundation for Statistical Computing, Vienna, Austria. URL https://www.R-project.org/ version 3.6.1.
6. SAS Institute Inc., 2011. The SAS system for Windows, Version 9.4., SAS Institute, Cary, NC.
7. SAS Institute Inc., 2018. JMP® Pro, Version 13.0., SAS Institute, Cary, NC.
8. Agresti A. 2013. Categorical Data Analysis, Third Edition. New York: Wiley.
9. Cox TF. 2005 An introduction to multivariate data analysis. London: Hodder Arnold.

B) Analytical Methods

Transcriptomics
Whole blood PAXgene RNA samples from the UK cohort were assayed by Illumina Human HT-12 v4 Expression BeadChip. Globin signal suppression was performed using the Affymetrix Globin Reduction protocol. The ASSESS data were derived from whole blood PAXgene RNA samples using a Clariom S Human transcriptome-wide gene expression profiling assay. Analysis of both arrays was performed using Bioconductor libraries in the R environment for statistical computing.

Serum Protein analysis
Serum κ-free light chain (FLC) and λ-FLC (Freelite®, The Binding Site Group Ltd), combined FLC (cFLC, Combylite™), and beta 2 microglobulin (β2M) on the SPAPLUS® turbidimeter (The Binding Site Group Ltd) were assayed following the manufacturer's recommendations. BAFF and CXCL13 were measured using a commercially available enzyme immunoassay (Human BAFF/BLyS/TNFSF13B and CXCL13/BLC/BCA-1 Quantikine ELISA Kits, R&D Systems, Abingdon UK) following the manufacturer's instructions. These data were derived from samples collected at the time of recruitment and are contemporaneous to the clinical data presented.

C) Supplementary figures

Supplementary Figure S1.

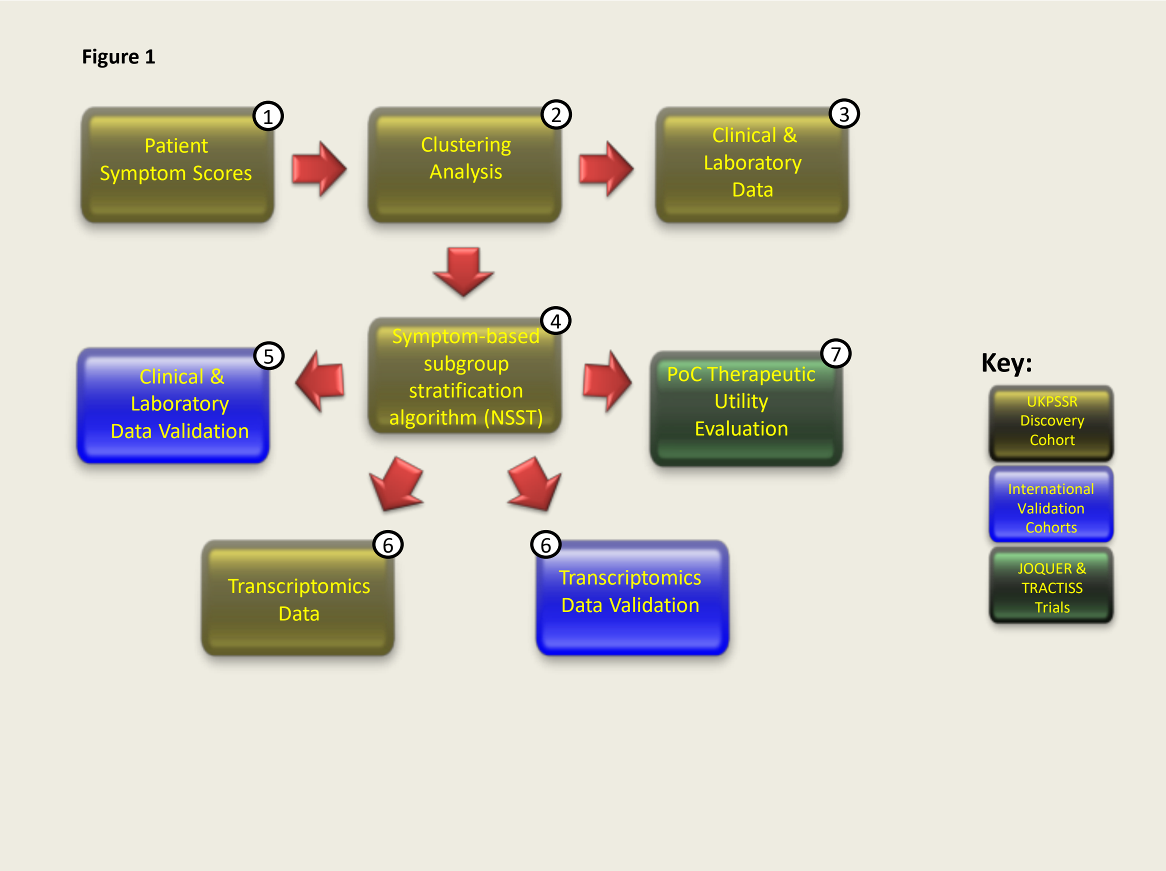

Supplementary Figure S1: Flow diagram showing stratification strategy and cohorts used for discovery, validation of biological findings in independent international cohorts, and evaluation of therapeutic utility. The UKPSSR cohort formed the Discovery Cohort (n=608) and the ASSESS (n=334) and Stavanger cohorts (n=62) were used as the external Validation Cohorts. Using patient reported symptom scores ① we identified four major clusters ②. Clusters showed marked differences in objectively measured clinical and laboratory data ③. We then created a stratification tool (NSST) permitting other cohorts to be stratified using this symptom-based stratification approach ④. We used the NSST to stratify patients in the ASSESS and Stavanger cohorts confirming the biological and clinical differences observed in the UKPSSR cohort ⑤. We observed also consistent differences in the transcriptomic profiles of our symptom-based subgroups across the UKPSSR (n=196) and the ASSESS cohorts (n=312) ⑥. The theragnostic potential of these findings is illustrated using the NSST ④ to stratify patients in two phase III clinical trials - JOQUER (n=107) and TRACTISS (n=114). We identified distinct subgroups responding respectively to hydroxychloroquine (HSB) and rituximab (DDF) treatments ⑦. The number of HSB patients in the JOQUER trial was 32 (hydroxychloroquine = 14 and placebo = 18). The number of DDF patients in the TRACTISS study was 26 (rituximab=13, placebo=13). See text for details.

Supplementary Figure S2A

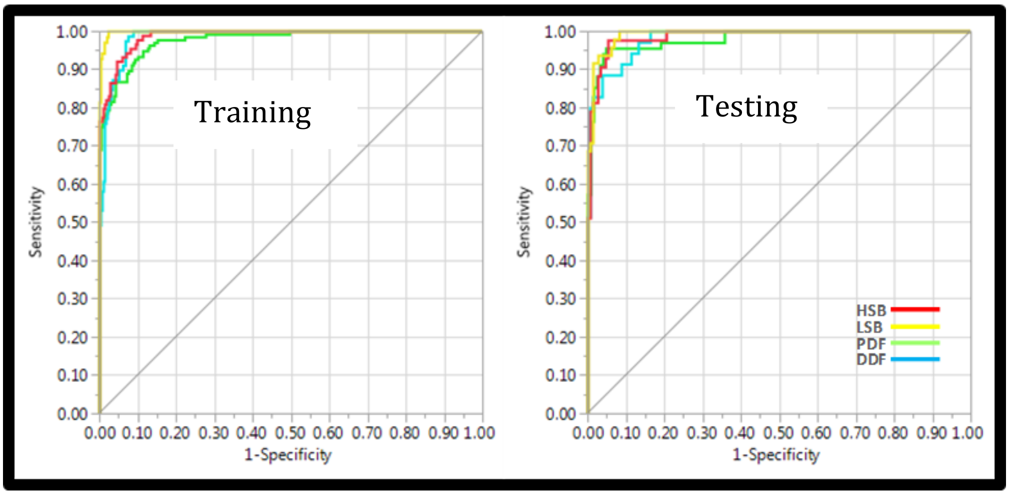

Subgroup	AUC ROC (95% CI)	
	Training set	Testing set
LSB	0.998 (0.993, 0.999)	0.992 (0.952, 0.999)
HSB	0.988 (0.974, 0.991)	0.987 (0.967, 0.999)
PDF	0.978 (0.963, 0.985)	0.980 (0.947, 0.996)
DDF	0.986 (0.970, 0.990)	0.981 (0.947, 0.996)

Supplementary Figure S2A: Receiver Operating Characteristic Curves for the Newcastle Sjögren's Stratification Tool (NSST) for the Training and Testing datasets. To develop a stratification tool permitting symptom-based subgroup membership to be identified based upon the patient reported symptom scores we fitted a multinomial logistic regression model to a randomly chosen training subset of 421 pSS patients from the UK discovery cohort. The Receiver Operating Characteristic (ROC) curve for this model is presented along with the corresponding ROC curve for the holdout testing dataset of the remaining one-third (187) patients. The sensitivity and specificity of the NSST is excellent with an Area Under the Curve (AUC) for the ROC curves >0.95 for all four symptom-based subgroups, indicating that this stratification tool faithfully represents the initial clustering-based approach. To permit other researchers to use this classification tool we provide an Excel macro of this stratification tool permitting pSS patients to be assigned to the **HSB**, **LSB**, **DDF** and **PDF** subgroups based on the severity of their five key symptoms.

Supplementary Figure S2B

Parameter estimates and standard errors for the multinomial logistic regression model (i.e. the NSST algorithm).

Term	Estimate	Std Error	ChiSquare	Prob>ChiSq
Intercept	-16.1987	3.2911	24.23	<.0001
DRYNESS	1.1345	0.3180	12.73	0.0004
FATIGUE	1.3927	0.3307	17.74	<.0001
PAIN	2.1199	0.4061	27.25	<.0001
ANXIETY	-0.0411	0.1381	0.09	0.766
DEPRESSION	0.2808	0.1980	2.01	0.1561
Intercept	-21.5221	3.5259	37.26	<.0001
DRYNESS	2.8911	0.4156	48.38	<.0001
FATIGUE	1.8563	0.3581	26.88	<.0001
PAIN	0.3212	0.4091	0.62	0.4323
ANXIETY	-0.3361	0.1580	4.52	0.0334
DEPRESSION	0.1750	0.2127	0.68	0.4106
Intercept	-31.9320	4.0985	60.7	<.0001
DRYNESS	1.6695	0.3478	23.05	<.0001
FATIGUE	1.4698	0.3593	16.73	<.0001
PAIN	1.6045	0.4232	14.37	0.0001
ANXIETY	0.7627	0.1782	18.32	<.0001
DEPRESSION	1.0337	0.2284	20.48	<.0001

For log odds of PDF/LSB, DDF/LSB, HSB/LSB

Supplementary Figure S3

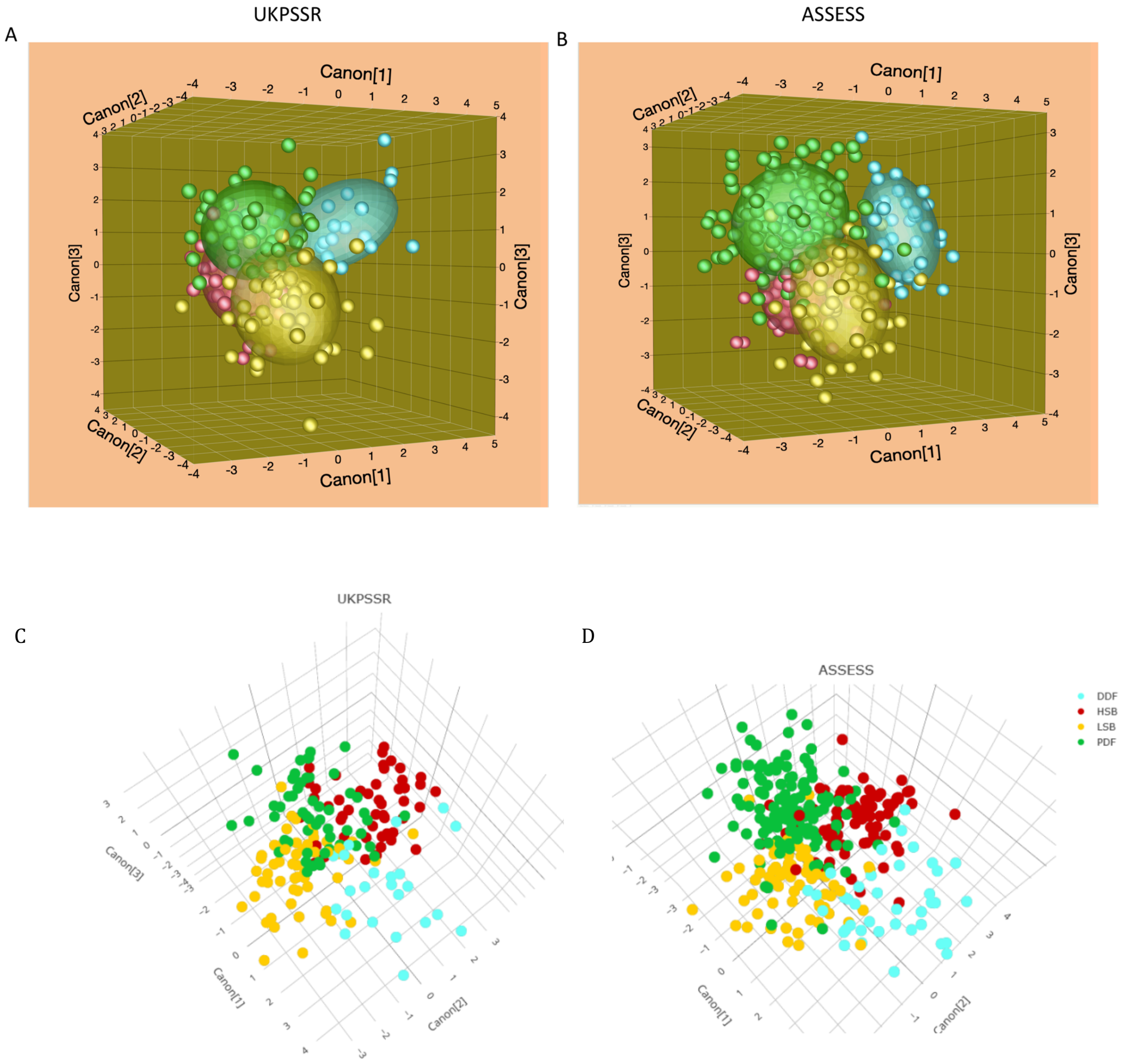

***Supplementary Figure S3*: Discriminant Analysis.** Projection of pSS patient data in canonical space following discriminant analysis of the four subgroups using annotated Chaussabel Module Activity Scores. Module Activity Scores are the mean expression of all genes in each module. The clouds or density ellipsoids contain 50% of observed data for each subgroup. **A**) Patients from the UKPSSR cohort are separated on the first three canonical variables, which represent a weighted combination of Chaussabel module activity scores. The four subgroups (**LSB** (yellow) and **DDF** (teal), **HSB** (red) and **PDF** (green)) are separated from each other despite some overlap groups. **B**) Patients from the **ASSESS** cohort are separated on the same three canonical variables. Patients from the four subgroups occupy similar spatial regions of canonical space. **C and D)** alternative visualizations of the same 3-dimensional plots from a different angle. These plots are also available as a rotating 3D movie.

Supplementary Figure S4:

NSST Excel Marco
https://github.com/SJOGRENS/Symptom-Based-Subgroups

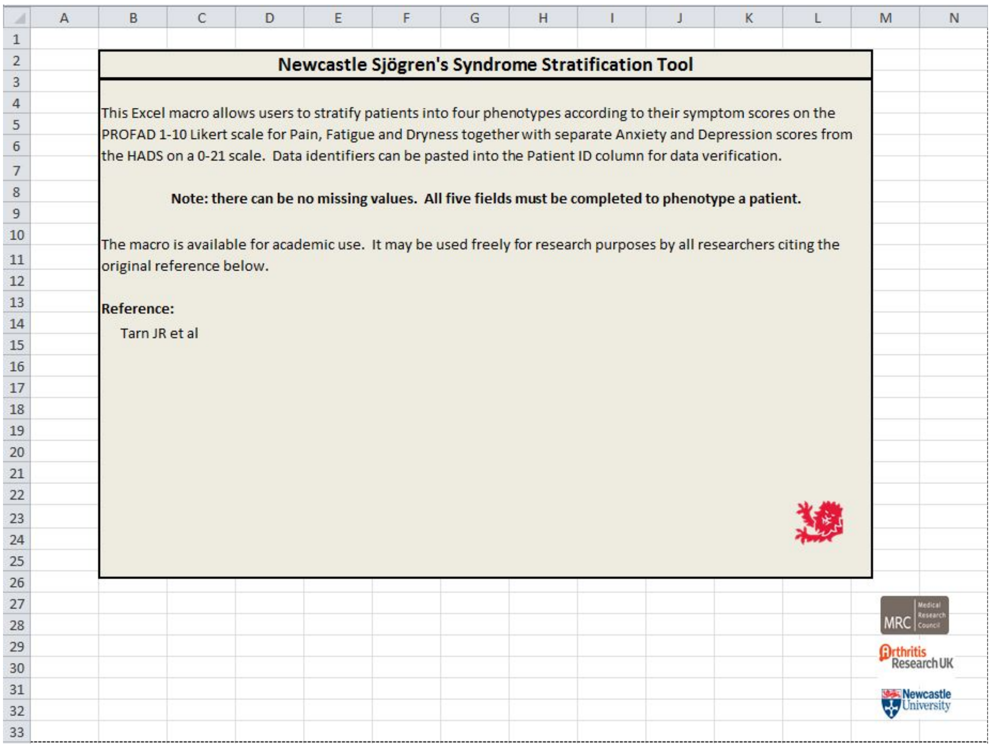

Supplementary Figure S4A: Newcastle Sjögren's Stratification Tool - Worksheet 1: Background and Permissions

Patient ID	PAIN	FATIGUE	DRYNESS	ANXIETY	DEPRESSION	Phenotype
1	10	9	10	21	20	HSB
2	1	1	1	0	0	LSB
3	1	9	9	1	1	DDF
4	3	9	3	1	1	PDF
5	1	1	1	1	3	LSB
6	1	1	9	1	1	DDF
7	3	3	3	3	3	LSB
8	1	9	9	1	9	DDF
9	9	3	1	1	9	PDF
10	3	1	3	3	9	LSB
11	1	1	6	3	9	LSB
12	5	5	9	3	9	DDF
13	5	5	5	5	5	PDF
14	5	5	5	5	5	PDF
15	5	5	1	19	19	HSB
16	1	1	1	1	1	LSB
17	1	1	1	8	21	HSB
18	9	9	9	9	9	PDF
19	1	1	1	1	1	LSB
20	9	9	9	19	19	HSB
21	1	1	1	1	1	LSB
22	1	1	1	1	1	LSB
23	9	9	9	16	16	HSB
24	9	9	9	1	15	PDF
25	9	1	1	9	14	PDF
26	9	9	9	13	13	HSB
27	9	9	9	12	12	HSB
28	3	3	3	3	3	LSB
29	9	9	9	10	10	HSB
30	1	1	1	1	1	LSB
31	9	9	9	8	8	PDF

***Supplementary Figure S4B*:** Newcastle Sjögren's Stratification Tool - Worksheet 2: Data Entry Sheet

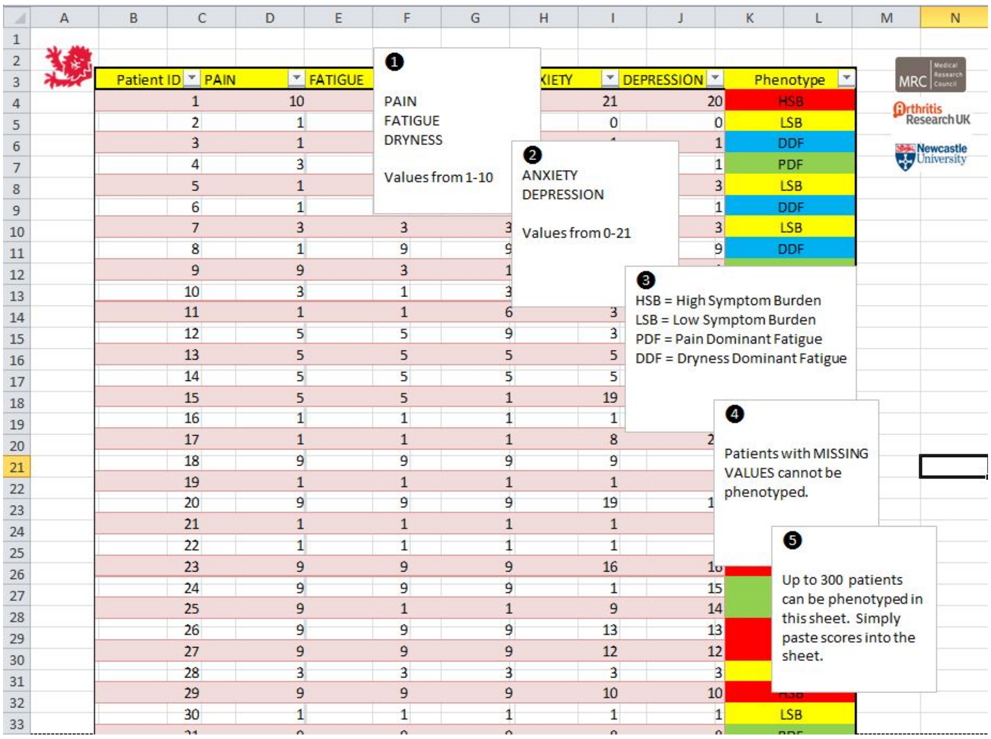

Supplementary Figure S4C: Newcastle Sjögren's Stratification Tool - Worksheet 3: User Guidance Notes

D) Supplementary Tables

Supplementary Table S1

**Parameter**	**UKPSSR**	**ASSESS**	**Stavanger**	**P-value**
Age (years)	60·8 (50·1, 67·8)	58.0 (50.0, 66.0)	62.0 (55·5, 71.0)	0·0664
Female (%)	95	93	82	0·0122
BMI (kg/m^2)	25·8 (22·7, 29·1)	23·4 (20·7, 27·2)	24·7 (22·8, 27·4)	<0·0001
AECG years	5 (2, 10)	5 (2, 10)	11 (6, 15)	<0·0001
Disease Activity ESSDAI	3 (1, 7)	3 (2, 8)	5 (1, 8)	0·4745
Symptom Scores ESSPRI	5·7 (3·7, 7.0)	5·3 (3·7, 6·8)	6 (4·4, 6·9)	0·7014
N	608	334	62	

Supplementary Table S1**: Key clinical, demographic and phenotypic data for the UKPSSR, ASSESS and Stavanger cohorts**. Except where indicated, summary statistics are presented as percentages or median values with 25th and 75th centile values in parentheses, and probability values are for the Kruskal-Wallis rank analysis. Age, sex, BMI, disease activity (ESSDAI) and symptom scores (ESSPRI) are shown for the three cohorts. All three cohorts were made up largely of females around sixty years of age, with an AECG diagnosis of between five and eleven years in duration, and with comparable disease activity scores and symptom scores.

Supplementary Table S2.1

Symptom-based Subgroups

Parameter	Cohorts	LSB	HSB	DDF	PDF	p-value
Unstimulated Salivary Flow (ml/15 mins)	UKPSSR	0.4 (0.0, 1.05)	0.2 (0.0, 1.0)	0.1 (0.0, 0·8)	0.3 (0.0, 1·2)	0·0294#
	ASSESS	0.3 (0.1, 1.7)	0.4 (0·1, 2·2)	0.0 (0.0, 0·2)	0.2 (0·1, 1·5)	<0·0001
	Stavanger	1.7 (0.4, 2·2)	0.8 (0·1, 2·5)	0.2 (0.0, 0·7)	0.9 (0.0, 1·7)	0·1212
	Combined	0.3 (0·1, 1·5)	0.3 (0.0, 1·3)	0.1 (0.0, 0.2)	0.3 (0·0, 1·4)	<0·0001
Schirmer's Test (mm/5 mins)	UKPSSR	3.0 (0.0, 6·8)	3.0 (0·5, 10·5)	2.0 (0.0, 6·5)	4.0 (.01, 10·5)	0·0295#
	ASSESS	5·3 (2.0, 15.0)	5·8 (2·3, 14·4)	7.0 (0.0, 13)	7·5 (3·5, 15)	0·2644
	Stavanger	7.0 (2·5, 13·6)	6·8 (2·8, 23·1)	1·5 (0.0, 4·5)	5·5 (2·5, 14·5)	0·0240
	Combined	3·9 (0·5, 9.0)	5.0 (1.0, 12·5)	2·3 (0.0, 7·1)	5.0 (2.0, 12·5)	<0·0001
Lymphocytes ($x10^9$/L)	UKPSSR	1·2 (1.0, 1·6)	1·5 (1·2, 1·8)	1·3 (1.0, 1·7)	1·3 (1·0, 1·7)	0·0009#
	ASSESS	1·3 (1.0, 1·8)	1·5 (1·1, 1·7)	1·2 (1.0, 1·6)	1·5 (1·1, 1·8)	0·0251
	Stavanger	1·4 (0·7, 1·7)	1·9 (1·6, 2·4)	1·2 (0·9, 1·4)	1·8 (1·3, 2.0)	0·0303
	Combined	1·3 (1.0, 1·6)	1·5 (1·2, 1·7)	1·2 (1.0, 1·7)	1·4 (1·1, 1·8)	<0·0001
IgG (mg/dL)	UKPSSR	18·0 (14·5, 22·9)	14·1 (11·1, 18·2)	16·6 (13.0, 20·9)	14·4 (11·1, 19·5)	0·0009#
	ASSESS	15·0 (12·3, 18·7)	12·8 (10·7, 16·7)	15·2 (11·1, 20·6)	12·5 (9·8, 16·1)	0·0028
	Stavanger	14·0 (10·7, 16.0)	11·1 (9·3, 11·9)	14·9 (12·4, 18)	11·7 (10·0, 13·1)	0·0054
	Combined	16·6 (13·2, 21·5)	13·4 (10·7, 17.0)	16.0 (12·4, 20·3)	13·1 (10·2, 17·7)	<0·0001
SSA and/or SSB positive (%)	UKPSSR	93	87	94	85	0·0476#
	ASSESS	70	59	74	54	0·0490
	Stavanger	90	60	100	59	0·0018
	Combined	85	75	89	72	<0·0001
Number & percentage (in brackets) of each subgroup in each cohort	UKPSSR	119 (19·6%)	208 (34·2%)	120 (19·7%)	222 (36·5%)	N_{UKPSSR} = 608
	ASSESS	64 (19·2%)	85 (25·5%)	43 (12·9%)	141 (42·3%)	N_{ASSESS} = 334
	Stavanger	10 (16·1%)	5 (8·1%)	18 (29·0%)	29 (46·8%)	$N_{STAVANGER}$ = 62
	Combined	193 (19·2%)	237 (23·6%)	181 (18·0%)	393 (39·1%)	N_{TOTAL} = 1004

Supplementary Table S2.2

Symptom-based Subgroup						
**Parameter**	**Cohorts**	**LSB**	**HSB**	**DDF**	**PDF**	**p-value**
κ-free light chains (mg/L)	UKPSSR<br>ASSESS	3·20±0·52<br>2·80±0·55	3·06±0·52<br>2·72±0·59	3·25±0·61<br>2·89±0·64	3·08±0·47<br>2·59±0·56	0·0524#<br>0·0106
λ-free light chains (mg/L)	UKPSSR<br>ASSESS	2·94±0·45<br>2·75±0·52	2·86±0·47<br>2·69±0·51	3·00±0·50<br>2·78±0·65	2·86±0·44<br>2·63±0·52	0·0631#<br>0·3375
β2-microglobulin (mg/L)	UKPSSR<br>ASSESS	1·34±0·22<br>1·20±0·22	1·30±0·24<br>1·16±0·19	1·38±0·27<br>1·25±0·24	1·32±0·24<br>1·12±0·20	0·0524#<br>0·0031
CXCL13 (pg/ml)	UKPSSR*<br>ASSESS	4·86±0·53<br>4·74±1·00	4·97±0·63<br>4·93±1·12	5·33±0·68<br>4·98±0·78	5·00±0·70<br>4·48±0·83	0·0547#<br>0·0010
Lymphoma prevalence (% in bracket)	UKPSSR	2/119 (1·6%)	12/208 (5·6%)	13/120 (11·2%)	6/222 (2·6%)	0·0294#

Supplementary tables S2.1 and S2.2 are reproduced from Tables 1 and 2 in the main manuscript with p values corrected for multiple comparisons using the Benjamini Hochberg method #. CXCL13 was only measured in a subset of the UKPSSR cohort*.

Supplementary Table S3

***Supplementary Table S3*: Top 31 modules with significant differences between subgroups in the combined UK and French cohorts.** Mean Chaussabel activity scores are shown for each subgroup (after mean centering). Significance values are from analysis of variance of Chaussabel module activity scores across the symptom-based subgroups. BH = Benjamini Hochberg multiplicity adjustment.

Chaussabel Module	mean DDF	mean HSB	mean LSB	mean PDF	p value	BH Adjusted p value
**M1.2IFNResponse**	0.15	-0.01	0.38	-0.06	0.0092	0.0769
**M3.3CellCycle**	0.02	-0.01	0.03	-0.01	0.0282	0.1046
**M3.4IFNResponse**	0.07	-0.01	0.19	-0.03	0.0018	0.0634
**M3.5ProteinSynthesis**	-0.06	-0.01	0.02	0.01	0.0159	0.0840
**M4.12CellCycle**	-0.11	-0.01	0.00	0.01	0.0076	0.0695
**M4.15CytotoxicityNKCells**	-0.06	-0.01	0.02	0.01	0.0068	0.0685
**M4.1TCells**	-0.13	0.00	0.01	0.01	0.0202	0.0876
**M4.7Cell_Cycle**	-0.10	-0.01	0.01	0.01	0.0254	0.0976
**M5.10MitochondriaProteasome**	-0.05	-0.01	0.02	0.00	0.0170	0.0840
**M5.11TCells**	-0.07	0.00	0.01	0.00	0.0042	0.0634
**M5.12IFNResponse**	0.05	-0.01	0.11	-0.02	0.0053	0.0634
**M5.4ProteinSynthesis**	-0.08	-0.02	0.02	0.01	0.0354	0.1220
**M5.5Apoptosis**	-0.07	-0.01	0.01	0.01	0.0115	0.0812
**M5.8TCells**	-0.05	0.00	0.02	0.00	0.0057	0.0634
**M6.15TCells**	-0.06	0.00	0.00	0.00	0.0227	0.0933
**M6.16Cell_Cycle**	-0.01	-0.01	0.03	-0.01	0.0048	0.0634
**M6.19TCells**	-0.07	0.00	0.00	0.02	0.0201	0.0876
**M6.2MitochondriaProteasome**	-0.03	0.00	0.02	0.00	0.0393	0.1267
**M6.7TCells**	-0.09	-0.01	0.02	0.01	0.0384	0.1267
**M6.9TCells**	-0.08	-0.01	0.01	0.01	0.0327	0.1167
**M7.12TCells**	-0.06	0.00	0.00	0.01	0.0165	0.0840
**M7.17TCells**	-0.06	0.00	0.01	0.01	0.0123	0.0812
**M7.21Monocytes**	0.00	0.00	0.04	-0.01	0.0111	0.0812
**M7.24TCells**	-0.04	0.00	0.01	0.00	0.0153	0.0840
**M7.25TCells**	-0.05	0.00	0.01	0.00	0.0021	0.0634
**M7.6TCells**	-0.05	-0.01	0.01	0.01	0.0130	0.0812
**M8.16LymphoidLineage**	-0.04	0.00	0.02	0.00	0.0044	0.0634
**M8.32BCells**	-0.07	0.00	0.01	0.01	0.0233	0.0933
**M8.68HematopoieticStemCells**	-0.06	0.00	0.03	0.00	0.0176	0.0840
**M8.86Erythropoiesis**	-0.08	0.00	0.03	0.01	0.0017	0.0634
**M8.90MatureBCells**	0.07	0.01	-0.02	-0.01	0.0044	0.0634

E) UKPSSR collaborators

WFN, SJB and BG are investigators of the UKPSSR. The other UKPSSR collaborators (as of 1 Jan 2017) include, in alphabetical order of their affiliations: Frances Hall (Addenbrooke's Hospital, Cambridge); Elalaine C Bacabac, Helen Frankland, Robert Moots (Aintree University Hospitals); Kuntal Chadravarty, Shamin Lamabadusuriya (Barking, Havering and Redbridge NHS Trust); Michele Bombardieri, Constantino Pitzalis, Nurhan Sutcliffe, Celia Breston (Bart and the London NHS Trust); Nagui Gendi, Karen Culfear (Basildon Hospital); Claire Riddell (Belfast Health and Social Care Trust); John Hamburger, Andrea Richards (Birmingham Dental Hospital); Saaeha Rauz (Birmingham & Midland Eye Centre); Sue Brailsford, Joanne Dasgin (Birmingham University Hospital); Joanne Logan, Diarmuid Mulherin (Cannock Chase Hospital); Jacqueline Andrews, Paul Emery, Alison McManus, Colin Pease, David Pickles (Chapel Allerton Hospital, Leeds); Alison Booth, Marian Regan (Derbyshire Royal Infirmary); Jon King, Amanda Holt (Derriford Hospital, Plymouth); Theodoros Dimitroulas, Lucy Kadiki, Daljit Kaur, George Kitas (Dudley Group of Hospitals NHS Foundation Trust); Abdul Khan, Tracey Cosier (East Kent Hospitals University); Panthakalam, Kelly Mintrim (East Sussex Healthcare NHS); Mark Lloyd, Lisa Moore (Frimley Park Hospital); Esther Gordon, Cathy Lawson (Harrogate District Foundation Trust Hospital); Monica Gupta, John Hunter, Lesley Stirton (Gartnavel General Hospital, Glasgow); Gill Ortiz, Elizabeth Price; Suzannah Pelger (Great Western Hospital); Claire Gorman, Balinder Hans (Homerton Hospital); Gavin Clunie, Suzanne Lane, Ginny Rose, Sue Cuckow (Ipswich Hospital NHS Trust); Michael Batley, Ruby Einosas (Maidstone Hospital); Susan Knight, Deborah Symmons, Beverley Jones (Macclesfield District General Hospital & Arthritis Research UK Epidemiology Unit, Manchester); Andrew Carr, Suzanne Edgar, Francisco Figuereido, Heather Foggo, Dennis Lendrem, Iain Macleod, Sheryl Mitchell, Christine Downie, Jessica Tarn, James Locke, Shereen Al-Ali, Sarah Legg, Kamran Mirza, Ben Hargreaves, Laura Hetherington (Newcastle upon Tyne Hospitals NHS Foundation Trust and Newcastle University); Adrian Jones, Peter Lanyon, Alice Muir (Nottingham University Hospital); Paula White, Steven Young-Min (Portsmouth Hospitals NHS Trust); Susan Pugmire, Saravanan Vadivelu (Queen's Elizabeth Hospital, Gateshead); Annie Cooper, Marianne Watkins (Royal Hampshire County Hospital); Anne Field, Stephen Kaye, Devesh Mewar, Patricia Medcalf, Pamela Tomlinson, Debbie Whiteside (Royal Liverpool University Hospital); Neil McHugh, John Pauling, Julie James, Andrea Dowden (Royal National Hospital for Rheumatic Diseases); Mohammed Akil, Jayne McDermott, Olivia Godia (Royal Sheffield Hospital); David Coady, Elizabeth Kidd, Lynne Palmer (Royal Sunderland Hospital); Charles Li (Royal Surrey Hospital); Sarah Bartrum, Dee Mead (Salisbury District Hospital); Bhaskar Dasgupta, Victoria Katsande, Pamela Long, Olivia Godia (Southend University Hospital); Erin Vermaak, Janet Turner (Staffordshire Stoke-on-Trent Partnership); Usha Chandra, Kirsten MacKay (Torbay Hospital); Stefano Fedele, Ada Ferenkeh-Koroma, Ian Giles, David Isenberg, Helena MaConnell, Nyarko Ahwireng, Stephen Porter (University College Hospital & Eastman Dental Institute); Paul Allcoat, John McLaren (Whyteman's Brae Hospital, Kirkaldy).

F) ASSESS collaborators

The authors thank all the investigators of the ASSESS cohort: Valerie Devauchelle-Pensec, Philippe Dieude, Jean Jacques Dubost, Anne-Laure Fauchais, Vincent Goeb, Eric Hachulla, Claire Larroche, Véronique Le Guern, Jacques Morel, Aleth Perdriger, Xavier Puéchal, Stephanie Rist, Damien Sene, Jean Sibilia, Olivier Vittecoq., The authors also thank Joelle Benessiano, Sarah Tubiana and all staff members from the Centre de Ressources Biologiques, Hôpital Bichat, AP-HP for their help in centralizing and managing biologic data collection from the French ASSESS cohort; Karine Inamo, Stanie Gaete, Djilali Batouche, Domitille Molinari from the Unité de Recherche Clinique Paris-Sud, AP-HP; and Mickael Randrianandrasana, Isabelle Pane, Adeline Abbe, Gabriel Baron, Philippe Ravaud from the Centre d'Epidémiologie clinique, Hôpital Hôtel Dieu, AP-HP, for clinical data collection and management.

G) JOQUER trial contributors

Jacques-Eric Gottenberg, Philippe Ravaud, Xavier Puéchal, Véronique Le Guern, Jean Sibilia,Vincent Goeb, Claire Larroche, Jean-Jacques Dubost, Stéphanie Rist, Alain Saraux, Valérie Devauchelle-Pensec, Jacques Morel, Gilles Hayem, Pierre Hatron, Aleth Perdriger, Damien Sene, Charles Zarnitsky, Djilali Batouche, Valérie Furlan, Joelle Benessiano, Elodie Perrodeau, Raphaele Seror, Xavier Mariette; JOQUER Trial Team

H) TRACTISS trial contributors

Brown S, Navarro Coy N, Pitzalis C, Emery P, Pavitt S, Gray J, Hulme C, Hall F, Busch R, Smith P, Dawson L, Bombardieri M, Ng W-F , Pease C, Price E, Sutcliffe N, Woods C, Ruddock S, Everett C, Reynolds C, Skinner E, Poveda-Gallego A, Rout J, Macleod I, Rauz S, Bowman S; TRACTISS Trial Team
